# Modification of Polysulfone Substrate with GO–PAMAM Nanocomposite for Improved Desalination Performance

**DOI:** 10.3390/membranes16030101

**Published:** 2026-03-10

**Authors:** Mohd Muzammil Zubair, Ahmed T. Yasir, Abdelbaki Benamor, Syed Javaid Zaidi

**Affiliations:** 1 UNESCO Chair in Desalination and Water Treatment, Center for Advanced Material, Qatar University, Doha P.O. Box 2713, Qatar; 2 Department of Mechanical and Industrial Engineering, Qatar University, Doha P.O. Box 2713, Qatar; 3 Department of Chemical and Hazardous Waste Management, Ministry of Environment and Climate Change, Doha P.O. Box 22332, Qatar; atyasir@mecc.gov.qa; 4 Gas Processing Centre, College of Engineering, Qatar University, Doha P.O. Box 2713, Qatar

**Keywords:** reverse osmosis, thin-film composite (TFC), graphene oxide, PAMAM, polysulfone substrate

## Abstract

Globally, freshwater scarcity is driving the urgent demand for advanced and new desalination technologies to overcome the shortage of clean water. Reverse osmosis (RO) membranes dominate seawater and brackish water treatment but are limited by the permeability–selectivity trade-off, fouling, and structural instability. To overcome these challenges, we employed a phase inversion process to fabricate polysulfone (PSF) supports embedded with a graphene oxide–poly(amidoamine) (GO-PAMAM) nanocomposite at three concentrations (0.03, 0.06, and 0.10 wt%), alongside a pristine control membrane with no GO-PAMAM. Systematic variation in GO-PAMAM loading revealed that a 0.06 wt% nanoparticle helps in producing a more uniform polyamide layer that achieves a high NaCl rejection (95.88%) and higher water flux (42.6 L m^−2^ h^−1^). The performance was evaluated at an operating pressure of 20 bar with a feed flow rate of 4 L min^−1^. The optimized membrane also demonstrated an improved fouling resistance, retaining 93% of its initial flux after fouling. This scalable approach highlights substrate-level modification as an effective strategy for next-generation RO membranes, advancing sustainable and energy-efficient desalination to meet escalating global water demands.

## 1. Introduction

The global scarcity of freshwater has emerged as one of the most pressing challenges of the twenty-first century, threatening human health, food security, industrial development, and environmental sustainability [1]. According to the United Nations, more than two billion people already live under conditions of severe water stress, and this number is projected to rise dramatically by 2050 [2,3]. These challenges are particularly acute in arid and semi-arid regions, where natural freshwater reserves are inherently limited. Therefore, there is an urgent need for sustainable and efficient technologies to produce clean water from unconventional sources such as seawater, brackish water, and reclaimed wastewater [4].

Desalination is increasingly regarded as a practical option for addressing freshwater scarcity [5]. In general, desalination technologies fall into two categories: thermal processes such as multi-stage flash (MSF) and multi-effect distillation (MED), and membrane-based separation methods [6]. While thermal desalination is well established, it is typically associated with high energy demand and operating costs. Membrane desalination, particularly reverse osmosis (RO), has therefore become the leading technology because it offers a higher scalability and comparatively lower energy consumption [7]. RO relies on a pressure-driven transport mechanism, where water permeates through a dense selective layer while dissolved salts are rejected [8]. Despite its widespread use, RO performance is still limited by the permeability–selectivity trade-off, meaning that gains in water permeability can come at the expense of salt rejection and long-term stability [9].

Modern RO membranes are usually made in the form of thin-film composites with a polyamide (PA) active layer formed on a porous polymer support with a non-woven backing [10,11]. PA membranes are usually made through the interfacial polymerization of m-phenylenediamine (MPD) and trimesoyl chloride (TMC) [12]. Most studies have concentrated on modifying the PA layer [13], although recent studies suggest that the role of the support in governing PA layer morphology and defect density cannot be ignored [14]. Support materials traditionally made of polysulfone (PSF) are known to be mechanically stable but hydrophobic in nature, which may cause defects in the PA layer through a funnel effect [15,16,17]. Substrate engineering is now recognized as a promising area in the development of thin-film composite membranes with better performance characteristics, although this area is relatively less explored [18].

To enhance substrate wettability and interfacial compatibility with the polyamide selective layer, substrate modification using nanomaterials has been widely explored as a scalable route to tune membrane microstructure and surface chemistry [19,20,21]. Graphene oxide has attracted particular interest because its two-dimensional framework and oxygen-containing functional groups improve hydrophilicity and promote interactions with polymer chains, contributing to improved transport and fouling-related performance [22,23,24]. Incorporating GO into membrane structures has been shown to enhance water flux, improve fouling resistance, and increase chlorine tolerance [25]. However, graphene oxide can aggregate in polymer casting solutions, limiting the uniformity of substrate modification [25,26,27]. Poly(amidoamine) (PAMAM) dendrimers provide complementary functionality through their highly branched architecture and amine-rich surface chemistry, which enhances hydrophilicity and can influence monomer uptake and interfacial polymerization behavior [28,29]. The covalent functionalization of graphene oxide with PAMAM yields a hybrid nanocomposite that improves dispersion stability and introduces additional reactive and hydrophilic sites, facilitating homogeneous incorporation within polymer supports and enabling a more effective substrate-controlled regulation of selective-layer formation [30].

**Figure 1:**
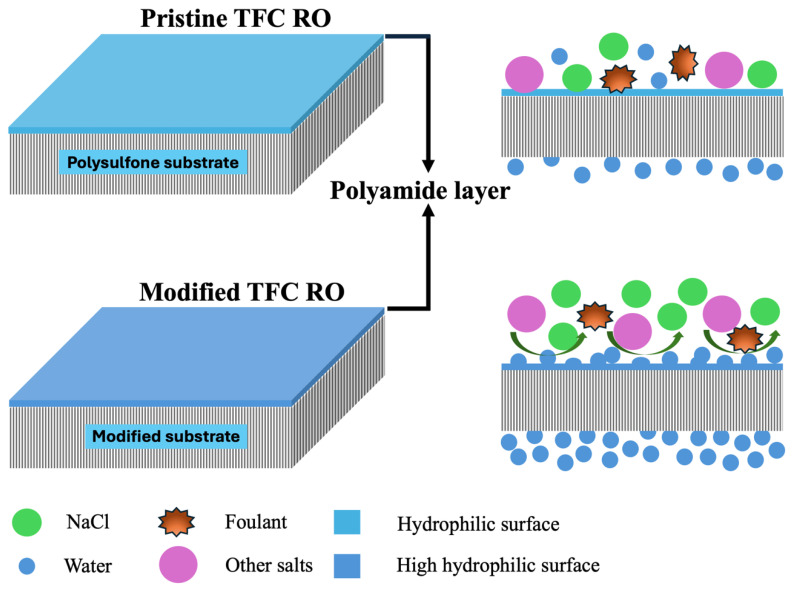
Schematic showing pristine vs. GO-PAMAM-modified TFC RO membranes, highlighting improved PA layer formation for water transport and fouling resistance with the modified substrate.

Despite the promise of graphene oxide and PAMAM, most previous studies have focused on incorporating these materials into the PA selective layer rather than modifying the porous support, leaving a key gap in substrate-level engineering. Substrate modification can regulate monomer uptake and transport pathways during interfacial polymerization and thereby influence PA morphology and performance [31]. In this work, GO–PAMAM nanocomposites were embedded into PSF supports to investigate their effect on selective-layer formation and RO desalination performance. Figure 1 schematically illustrates the difference between the pristine TFC membrane and the modified design.

Four TFC membranes were fabricated: a pristine support (M0) and GO–PAMAM-modified supports at 0.03 wt% (M1), 0.06 wt% (M2), and 0.10 wt% (M3), followed by PA formation via MPD/TMC interfacial polymerization. Membranes were characterized using SEM, AFM, FTIR, and contact angle measurements, and evaluated through water flux and salt rejection. The results show that a moderate loading (0.06 wt%) promotes the formation of a more uniform PA layer and improved desalination performance, whereas a higher loading leads to aggregation-induced structural non-uniformity and reduced performance. Overall, the study highlights substrate engineering using GO–PAMAM as a practical route to tune PA formation and improve TFC RO membrane performance.

## 2. Materials and Methods

### 2.1. Materials

The materials used in this study mainly include the chemicals and reagents employed for the fabrication of the polysulfone membrane and for interfacial polymerization. Polysulfone (Mw ≈ 35 KDa), obtained from Sigma-Aldrich (St. Louis, MO, USA), was used to prepare the membrane support. A nonwoven polyester fabric (97 ± 10 µm thickness) was obtained from Wellspring Co., Ltd. (Gwangju City, South Korea). Second-generation poly(amidoamine) (PAMAM G2, 99.9%) was obtained from Dendritech, Inc (Midland, MI, USA). Extra-pure graphite powder (99.9%), sodium nitrate (NaNO_3_, 99.9%), potassium permanganate (KMnO_4_, 99.9%), sulfuric acid (H_2_SO_4_, 98 wt%), N,N-dimethylformamide (DMF, 99.9%), and bovine serum albumin (BSA, 99.9%) were obtained from Sigma-Aldrich. Trimesoyl chloride (TMC) was purchased from TCI Co., Ltd. (Tokyo, Japan), and m-phenylenediamine (MPD) from Sigma-Aldrich (St. Louis, MO, USA). Sodium sulfate (Na_2_SO_4_), magnesium chloride (MgCl_2_), sodium chloride (NaCl), and calcium chloride (CaCl_2_) (each >99.5%) were obtained from Merck (Darmstadt, Germany) and used to prepare 2000 ppm salt solutions. All chemicals were reagent grade or higher and used as received; distilled water was used throughout the experiments.

### 2.2. Synthesis of Graphene Oxide

GO was prepared using a modified Hummer’s method as reported by Mahmoudi et al. [32,33]. First, 2.5 g of NaNO_3_ and 5 g of graphite powder were dispersed in 115 mL of concentrated H_2_SO_4_ in a round-bottom flask and stirred in an ice bath for 30 min to maintain the temperature near 10 °C. Subsequently, 15 g of KMnO_4_ was slowly added over 2 h to control the reaction rate and heat generation. The mixture was then heated to 35 °C and maintained for 1 h to facilitate oxidation. Afterward, 230 mL of distilled water was carefully introduced while keeping the temperature below 100 °C, followed by stirring for an additional hour. A further 300 mL of distilled water was added, and finally 10 mL of 30% H_2_O_2_ was introduced to terminate the reaction, indicated by a distinct color change to bright yellow. The resulting product was purified through repeated centrifugation and washing with dilute HCl to remove residual metal ions and byproducts. The purified material was then freeze-dried to obtain fine, dry powder for subsequent membrane fabrication.

### 2.3. Synthesis of GO-PAMAM Composite

**Figure 2:**
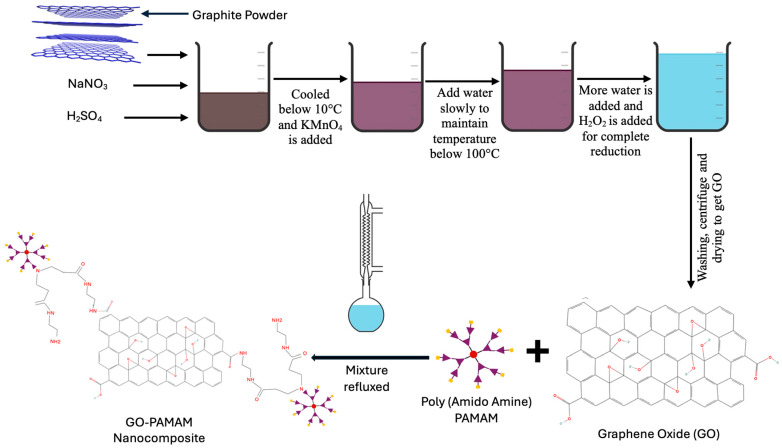
Schematic of GO-PAMAM synthesis process.

Generation 0 PAMAM dendrimers were grafted onto GO using the procedure reported by Rafi et al. [34], with slight modifications. In this process, 0.3 mg of GO was dispersed in 37.5 mL of DMF using continuous stirring to ensure complete exfoliation. Separately, 1.5 mg of G0 PAMAM was dissolved in 6 mL of methanol and then added dropwise to the GO dispersion under stirring. This combined mix was refluxed for 24 h at 80 °C to allow the functionalization reaction between GO and PAMAM. At the end of functionalization, the hot mixture was centrifuged twice with ethanol to remove unreacted materials and then washed three times with distilled water to remove any remaining impurities. It was then dried in the oven at 80 °C for 6 h to obtain a GO-G0 PAMAM nanocomposite, which was stored in a desiccator for further use. Higher generations of PAMAM (G2) were separately put through the same procedure and reacted with GO to obtain a series of functionalized nanocomposites. The stepwise production of GO-PAMAM nanocomposites will be represented in a schematic form in Figure 2.

### 2.4. Preparation of Membranes

Porous PSF substrates were prepared via non-solvent induced phase separation (NIPS) using DMF as the solvent [35]. The casting solution in the preparation contained 17.5 wt% PSF and GO-PAMAM at 0.03, 0.06, or 0.10% wt% concentration. First, the GO-PAMAM was dispersed in DMF and sonicated for 1 h, after which the PSF granules were added gradually under stirring to fully dissolve the polymer at 60 °C. After all the PSF granules were dissolved into the solution, stirring was continued for 6 h to homogenize the solution, then rested without stirring for 4 h to release any entrapped air bubbles.

The dope solution was than casted into a non-woven polyester fabric (thickness 97 ± 10 µm) using a casting knife of thickness 200 µm, and then immersed into a coagulation water bath at ambient temperature. Upon phase inversion, DMF diffused into the water; however, water also penetrated into the polymer film, through which it developed an asymmetric porous structure firmly bonded to the fabric backing. The membranes were kept submerged in distilled water for 24 h to leach out any residual solvent and favor pore stabilization.

TFC membranes were fabricated through IP on the prepared PSF substrates. The aqueous phase contained 3 wt% MPD [4] and 0.2 wt% SDS [36,37], the latter serving as a surfactant to improve substrate wetting and uniform MPD distribution. The organic phase consisted of 0.1 wt% TMC dissolved in *n*-hexane. The substrate was clamped in a rectangular frame, and 25 mL of the MPD/SDS solution was poured on top and left for 4 min before excess solution was removed using a rubber roller. Subsequently, 25 mL of TMC solution was poured over the surface and allowed to react for 50 s to form a highly cross-linked PA selective layer. Residual TMC was drained, and the membrane was thermally cured at 70 °C for 10 min to stabilize the PA layer [38].

**Figure 3:**
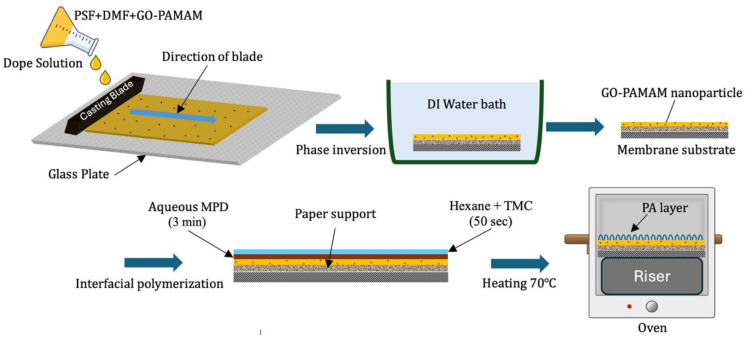
Step-by-step synthesis of nanocomposite TFC membrane.

The fabricated TFC membranes were thoroughly rinsed with deionized water to remove any unreacted chemicals and residual solvent. After rinsing, the membranes were stored in DI water and directly used for subsequent characterization and performance evaluation. A schematic representation of the complete fabrication process is shown in Figure 3, and the detailed compositions of the prepared membranes are summarized in Table 1.

### 2.5. Permeation and Retention Capacity of Prepared Membranes

**Figure 4:**
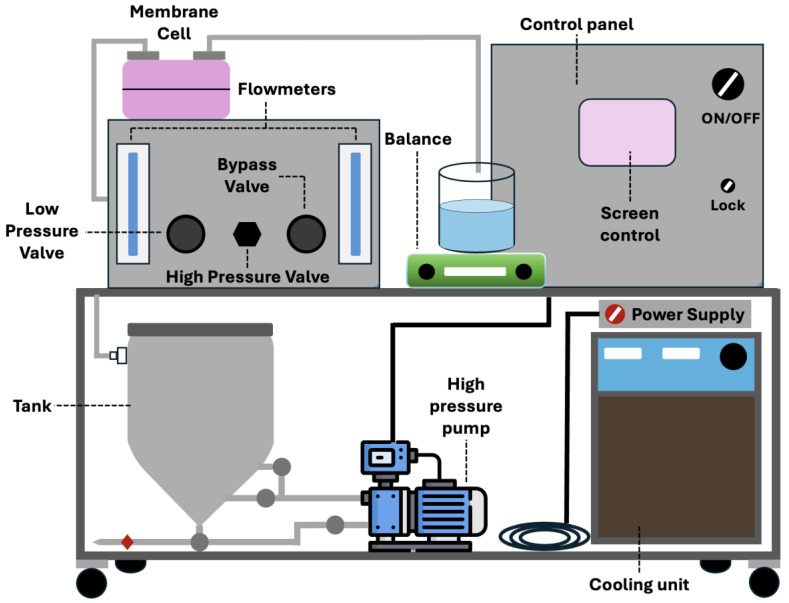
Schematic of the lab-scale crossflow membrane filtration system.

The desalination performance of the membranes was tested using a bench-scale cross-flow RO unit from Sterlitech Corporation (Auburn, WA, USA), as illustrated in Figure 4. This system was chosen to accurately assess how adding GO-PAMAM to the PSF support layer affects water transport and salt rejection. Each membrane sample was cut to an effective active area of 42 cm^2^ and carefully placed in the test cell to ensure a leak-free seal. Before testing, the membranes went through a compaction step. This involved circulating deionized (DI) water at 20 bar for 1 h, which helped stabilize the structure and reduce changes in initial water flux caused by the membrane’s mechanical relaxation.

The feed solution was made with DI water and sodium chloride (NaCl) at a concentration of 2000 ppm, which mimics a brackish water environment [39]. All experiments took place at room temperature with a constant pressure of 20 bar and a feed flow rate of 4 L/min. The pH of the feedwater was kept close to neutral without any adjustments. During each run, we collected the permeate at set time intervals in a calibrated cylinder to measure the volume of water passing through the membrane over time. We monitored the salt concentrations in both the feed and permeate streams using a digital conductivity meter (Hach Company, Loveland, CO, USA) to assess separation performance accurately.

The water flux (*J*_w_) of the membranes was calculated using Equation (1) [40,41]:

(1) $$J_{w}=\frac{∆V}{A_{m}\cdot ∆t}=A_{w}\cdot ∆P$$

where Δ*V* is the volume of permeate collected (L), *A_m_* is the effective membrane area (m^2^), and Δ*t* is the time of collection (h). The intrinsic water transport property was evaluated through the water permeability coefficient (*A_w_*), calculated from the applied transmembrane pressure (Δ*P*) in bar.

The salt rejection (*R*) was calculated using Equation (2), which compares the salt concentration in the permeate to that of the feed:

(2) $$R=\left(1-\frac{C_{p}}{C_{f}}\right)\times 100$$

where *C_f_* and *C_p_* are the salt concentrations in the feed and permeate streams (ppm), respectively. A higher rejection value reflects a more selective membrane, effectively preventing salt passage.

In addition to water and salt transport, the salt permeability (*B*) was calculated to assess the ability of the polyamide selective layer to block dissolved salts while allowing water to pass, using Equation (3) [40]:

(3) $$B=\left(\frac{1}{R}-1\right)\times J_{w}\;$$

where *R* is expressed as a decimal fraction and *J* is the water flux.

### 2.6. Antifouling Performance of the Prepared Membrane

The antifouling behavior of the prepared membranes was investigated using a 500 ppm BSA solution as the foulant [42]. The experiment was initiated by circulating DI water for 60 min to establish a stable baseline flux and ensure membrane stabilization prior to fouling assessment. After this period, the feed was switched to the BSA solution, and the system was operated for an additional 190 min, giving a total run time of 250 min.

The tests were conducted at an operating pressure of 10 bar, a feed flow rate of 2 L/min, and ambient temperature (~25 °C). These parameters were selected to generate hydrodynamic conditions that promote foulant deposition on the membrane surface while closely mimicking realistic RO operation [43]. Throughout the experiment, permeate flux was recorded at 10 min intervals to monitor the rate of flux decline as fouling progressed. A gradual decrease in flux was expected as organic matter accumulated on the polyamide surface, increasing hydraulic resistance to water transport.

Following the fouling cycle, the BSA solution was replaced with DI water and circulated at the same cross-flow rate for 60 min without applied transmembrane pressure to clean the membranes. This hydraulic cleaning step helped remove loosely bound foulants without chemical cleaning agents. The pure water flux was then measured again under identical conditions to determine the extent of performance recovery and to distinguish between reversible and irreversible fouling.

The normalized permeate flux was calculated using

(4) $$J_{n}\left(t\right)=\frac{J(t)}{J_{0}}$$

where *J_n_*(*t*) is the normalized flux at time t, *J*(*t*) is the instantaneous permeate flux, and *J*_0_ is the initial pure water flux measured before fouling.

To compare the overall fouling behavior of different membrane samples, an average normalized flux trend was calculated as

(5) $$J_{n,avg}\left(t\right)=\frac{J_{n,M0}\left(t\right)+J_{n,M2}(t)}{2}$$

where *J_n_*_,*M*0_(*t*) and *J_n_*_,*M*2_(*t*) represent the normalized flux values of membranes M0 and M2, respectively at time *t*. This averaged curve is used to illustrate the overall fouling trend of the membranes.

## 3. Results and Discussion

### 3.1. Characterization of Nanoparticle

#### 3.1.1. FTIR Analysis

**Figure 5:**
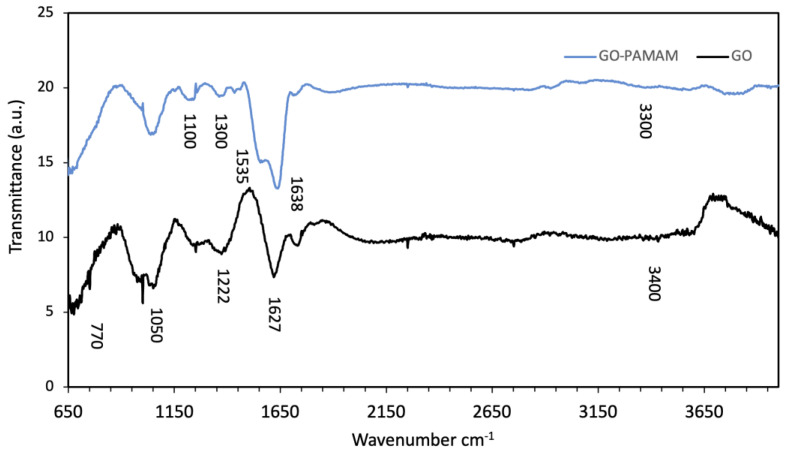
FTIR spectrum of synthesized GO and GO-PAMAM.

The Fourier-transform infrared (FTIR) spectra of pristine GO and the GO–PAMAM nanocomposite are presented in Figure 5. The FTIR spectra of synthesized GO and PAMAM-functionalized GO are shown in Figure 5. The GO spectrum revealed several absorption bands around 3400, 1627 and 1222 cm^−1^, which correspond to O–H stretching vibration, C=C skeletal vibration and C–O stretching of epoxy and carboxyl groups, respectively. Moreover, the absorption bands around 1050 and 770 cm^−1^ correspond to C–O stretching and aromatic C–H bending vibrations [44].

The GO–PAMAM nanocomposite showed additional peaks; the band between 1100 and 1300 cm^−1^ corresponds to C–N stretching vibration, the band around 1535 cm^−1^ corresponds to bending vibrations of N–H for amide II and stretching of C–N, the band around 1638 cm^−1^ corresponds to C=O stretching of amide I, and the band around 3300 cm^−1^ corresponds to the stretching vibration of N–H of amine and amide groups [45]. The presence of these absorption bands indicates the introduction of amine and amide containing PAMAM on the surface of GO.

#### 3.1.2. XRD Analysis

**Figure 6:**
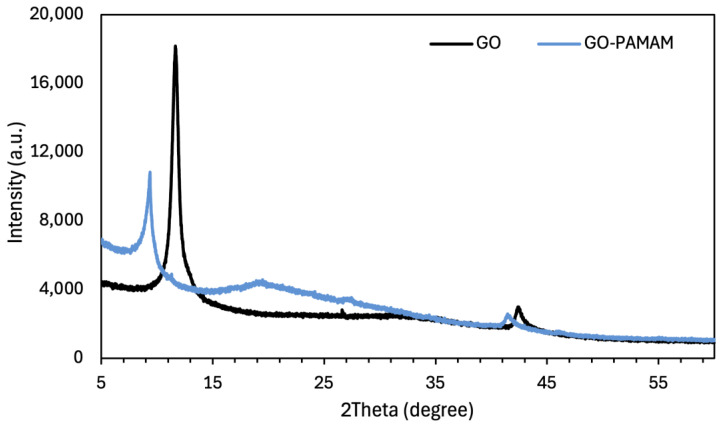
XRD spectrum of GO and GO-PAMAM.

The X-ray diffraction (XRD) patterns of GO and GO–PAMAM nanocomposites are presented in Figure 6. The GO sample exhibits a diffraction peak at 2θ ≈ 11.7°, corresponding to the (001) reflection plane. This peak indicates an increase in interlayer spacing associated with the introduction of oxygen-containing functional groups, including hydroxyl, epoxy, and carboxyl groups, as well as intercalated water molecules formed during the oxidation procedure [46]. Furthermore, the absence of the graphite (002) diffraction peak near 2θ ≈ 26° provides additional evidence for the oxidation of graphite and the formation of GO sheets.

After PAMAM functionalization, the GO (001) peak at 2θ ≈ 11.7° shifts to a lower diffraction angle of approximately 9.3° in the GO–PAMAM nanocomposite. The observed decrease in 2θ values corresponds to an increase in interlayer spacing from approximately 7.5 Å to 9.4 Å, which is attributed to the intercalation of PAMAM molecules between GO nanosheets [47]. This increase in interlayer spacing alters the stacking arrangement of the GO layers and decreases interlayer interactions between adjacent sheets. In addition, a diffuse scattering band in the range of 15–30° is observed, indicating changes in the stacking arrangement after functionalization.

### 3.2. Substrate FTIR Characterization

**Figure 7:**
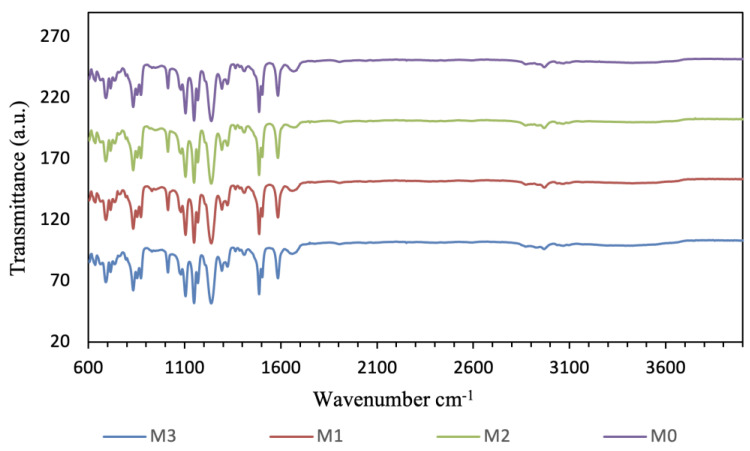
FTIR spectra of pristine and GO-PAMAM-modified PSF substrate (M0–M3).

Figure 7 shows the FTIR spectra of the pristine PSF support (M0) and GO–PAMAM incorporated PSF supports (M1–M3). PSF supports exhibit the characteristic absorption bands of polysulfone, including the sulfone group (O=S=O) stretching vibrations at approximately 1149–1150 cm^−1^ and 1290–1330 cm^−1^, as well as ether-related C–O–C stretching bands around 1110 and 1240 cm^−1^. In addition, the bands in the range of 1480–1600 cm^−1^ are attributed to the aromatic ring vibrations of the PSF backbone. These peaks confirm the successful formation and chemical stability of the PSF support layer after phase inversion. Compared to the pristine support, the GO–PAMAM-modified supports exhibit no major peak shifts or new dominant bands, which is expected due to the low loading of GO–PAMAM and the overlap of its functional group bands with the strong PSF absorption peaks. Overall, FTIR indicates that GO–PAMAM incorporation does not alter the PSF backbone and is primarily achieved by physical dispersion within the support matrix.

### 3.3. SEM Analysis of the Prepared TFC Membrane

**Figure 8:**
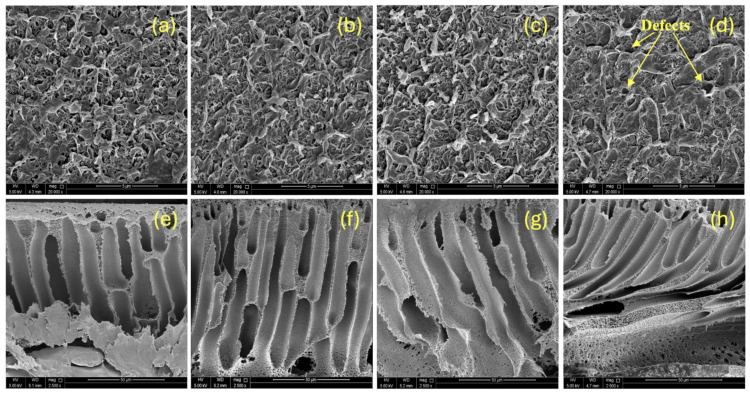
SEM images of surface (**a**–**d**) and cross-sectional (**e**–**h**) morphologies of pristine (M0) and GO-PAMAM-modified TFC membranes: (**a**,**e**) M0, (**b**,**f**) 0.03 wt% (M1), (**c**,**g**) 0.06 wt% (M2), and (**d**,**h**) 0.10 wt% (M3).

The surface and cross-sectional shapes of the pristine and GO-PAMAM-modified TFC membranes were studied using scanning electron microscopy (SEM) to understand how nanoparticle addition affects membrane formation (Figure 8). The top images (Figure 8a–d) show the PA selective layer, and the bottom images (Figure 8e–h) display the asymmetric cross-sections of the PSF support layer.

The pristine membrane (M0, Figure 8a) exhibited the typical ridge-and-valley morphology of PA films formed through the IP of MPD and TMC. This uneven nodular structure, resulting from rapid diffusion-limited polymerization, increases the effective surface area but also creates potential fouling sites. Furthermore, the morphology was not uniform across the entire membrane surface. Adding 0.03 wt% GO-PAMAM (M1, Figure 8b) resulted in a more even and compact surface. This suggests better MPD diffusion and controlled nucleation. At 0.06 wt% loading (M2, Figure 8c), the PA layer appeared dense and well connected, indicating a defect-free selective layer optimized for salt rejection. However, at 0.10 wt% (M3, Figure 8d), nanoparticle clustering disrupted uniform growth, leading to micro-defects that reduced membrane selectivity.

Cross-sectional images confirmed the standard asymmetric structure of TFC membranes. The pristine support (M0, Figure 8e) showed narrow finger-like macrovoids that limited water transport. With 0.03 wt% GO-PAMAM (M1, Figure 8f), macrovoids became more open and elongated. At 0.06 wt% GO-PAMAM (M2, Figure 8g), the support layer exhibited well-organized and enlarged vertical channels, which facilitate efficient water transport. At 0.10 wt% (M3, Figure 8h), structural distortion from nanoparticle clustering interfered with proper phase inversion.

### 3.4. Contact Angle Analysis of the Prepared TFC Membranes

**Figure 9:**
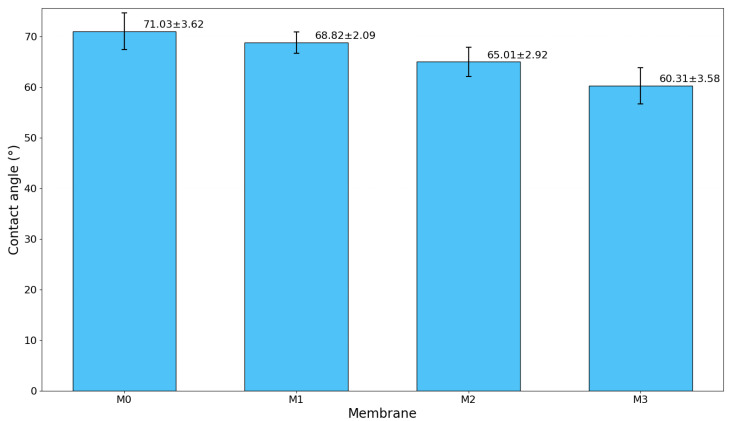
Contact angle of the synthesized membrane.

The surface wettability of both pristine and GO-PAMAM-modified TFC membranes was assessed using contact angle measurements (Figure 9). The pristine membrane (M0) showed the highest contact angle of 71.03 ± 3.62°, indicating its relatively hydrophobic nature stemming from the unmodified polysulfone support and polyamide selective layer. This hydrophobicity restricts interactions between water and the membrane, which lowers water permeability and raises the concern for membrane fouling [48]. Incorporating GO-PAMAM nanoparticles led to a gradual decrease in contact angle, indicating improved surface hydrophilicity. At 0.03 wt% (M1), the contact angle dropped to 68.82 ± 2.09°. This change is likely due to the addition of hydrophilic functional groups like hydroxyl (-OH) and amine (-NH_2_) from GO-PAMAM. A further decrease to 65.01 ± 2.92° at 0.06 wt% (M2) suggests a more even dispersion of nanoparticles. This better distribution likely helps create a stable hydration layer on the membrane surface. As a result, water–surface interactions may improve, which could reduce the adhesion of foulants. Given that this membrane is highly hydrophilic, it makes sense that greater surface roughness encourages water molecules to spread, leading to a lower contact angle, which aligns with wetting theory [49].

At the highest loading, 0.10 wt% (M3), the contact angle dropped to 60.31 ± 3.58°. However, despite the increased hydrophilicity, excessive GO–PAMAM incorporation may promote nanoparticle agglomeration. This agglomeration can potentially introduce structural defects or non-selective voids within the membrane matrix, which may negatively influence salt rejection and long-term membrane stability.

### 3.5. AFM Analysis of the Prepared TFC Membranes

**Figure 10:**
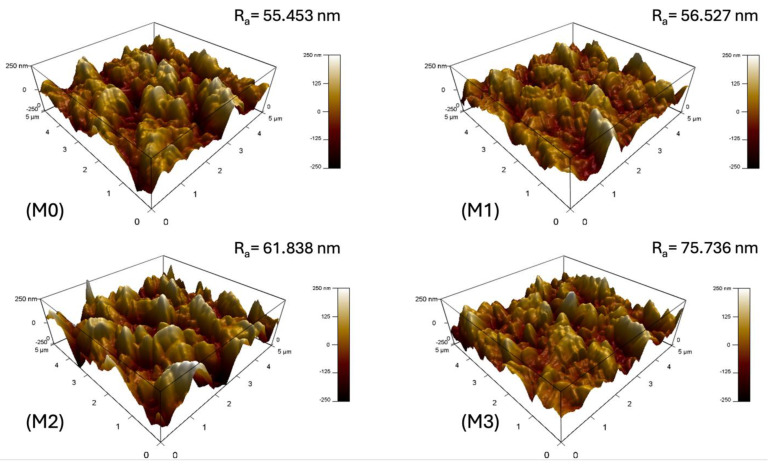
AFM analysis of the prepared membrane.

The surface roughness of the pristine and GO-PAMAM-modified TFC membranes was analyzed using AFM to see how adding nanoparticles affected the PA active layer. In the AFM images shown in Figure 10, bright areas represent peaks of the surface, while darker areas show valleys and pores in the membrane. The pristine membrane (M0) had a typical ridge-and-valley structure with a mean roughness (Ra) of 55.45 nm, which is common for PA layers made through IP. Adding a small amount of GO-PAMAM (0.03 wt%, M1) slightly increased the surface roughness to 56.53 nm. This change implies better nucleation during the formation of the polyamide, but it did not disrupt the layer’s uniformity. Increasing the nanoparticle concentration to 0.06 wt% (M2) caused a more significant rise in roughness to 61.83 nm. This change also resulted in a well-connected, textured structure that helps boost the effective surface area and improve water transport, as noted in previous studies on membranes [50].

Meanwhile, for the highest GO-PAMAM loading of 0.10 wt% (M3), the roughness rose to 75.73 nm, showing irregular and disordered surface features. This behavior is due to the larger molecular size and branched structure of PAMAM. These properties lead to nanoparticle aggregation and disrupt uniform IP, similar to what Li et al. [51] observed. While moderate surface roughness can improve permeability by increasing the active area, too much roughness creates deep valleys and sharp peaks. These features serve as spots for foulants to cling, which speeds up foulant formation and reduces long-term stability. This shows the need to fine-tune nanoparticle concentration to find a balance between permeability and fouling resistance.

### 3.6. Reverse Osmosis Performance of Prepared Membranes

**Figure 11:**
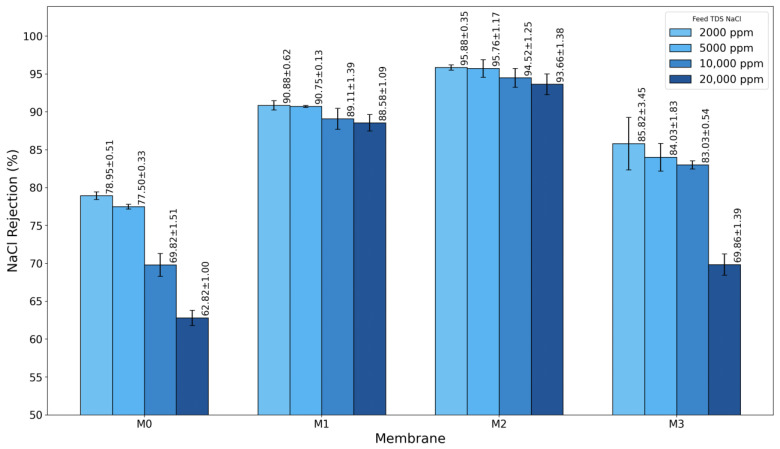
Salt rejection of different membranes at varying feed concentrations (2000, 5000, 10,000, and 20,000 ppm NaCl).

**Figure 12:**
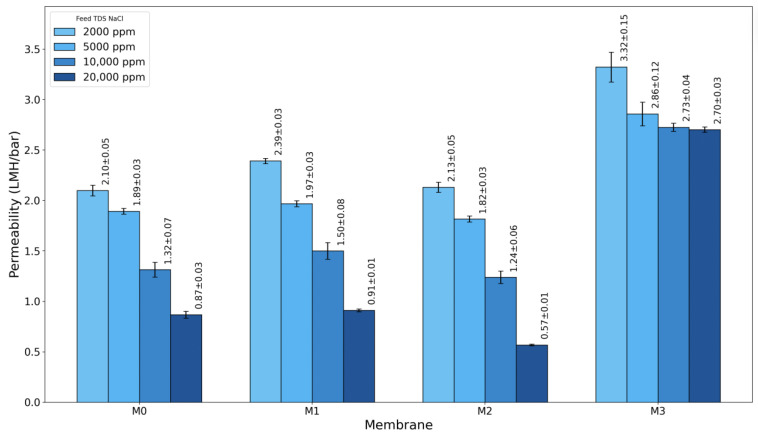
Water permeability of membrane (M0, M1, M2, M3) at different NaCl feed concentrations (2000, 5000, 10,000, and 20,000 ppm).

**Figure 13:**
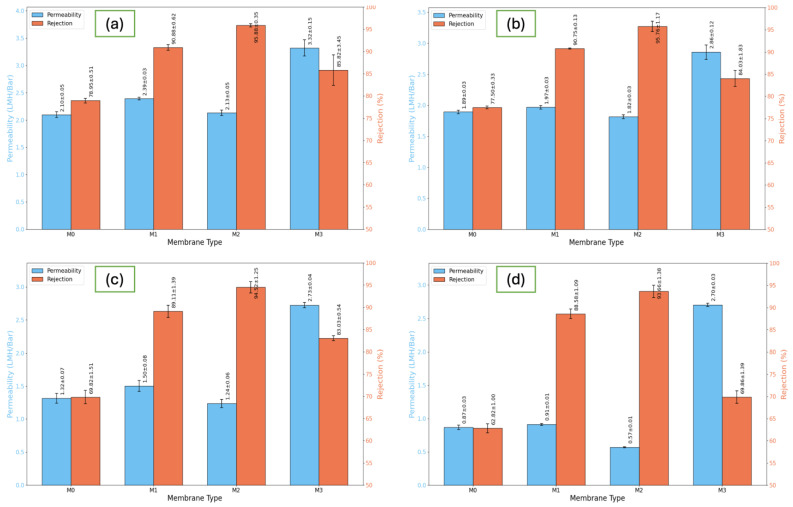
Combined NaCl rejection and water permeability of (M0, M1, M2, M3) TFC membranes at different feed concentrations (NaCl), with (**a**) for 2000 ppm, (**b**) for 5000 ppm, (**c**) for 10,000 ppm, and (**d**) for 20,000 ppm.

**Figure 14:**
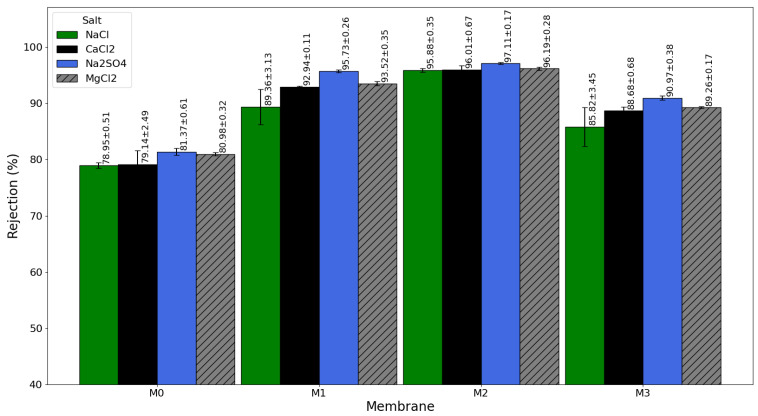
Salt rejection performance of pristine (M0) and GO-PAMAM-modified TFC membranes (M1, M2, M3) for different salts (NaCl, CaCl_2_, MgCl_2_, and Na_2_SO_4_).

**Figure 15:**
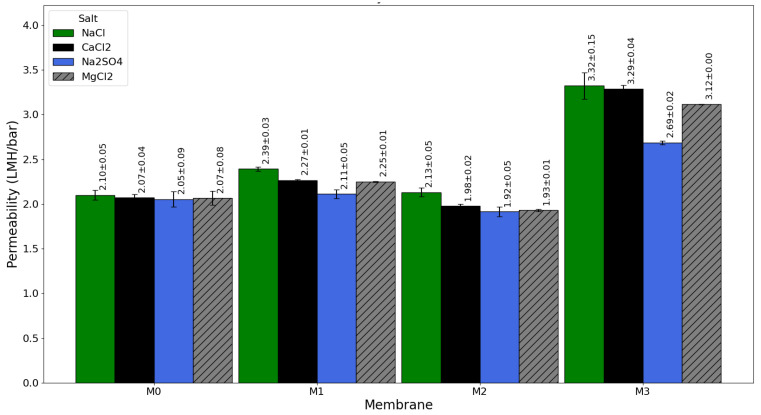
Water permeability of pristine (M0) and GO-PAMAM-modified TFC membranes (M1, M2, M3) tested with different salts.

**Figure 16:**
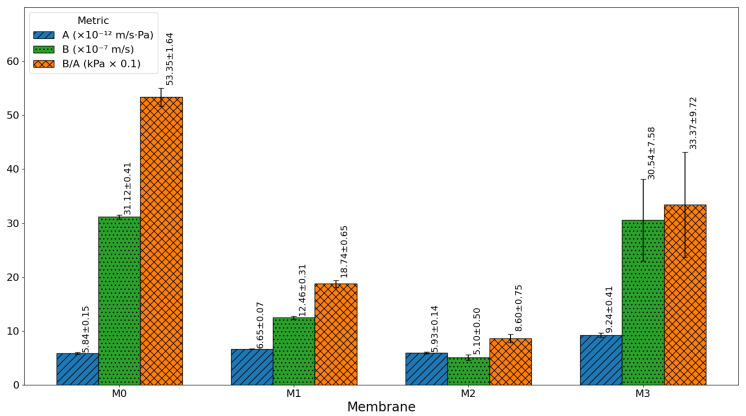
Separation characteristics for NaCl.

**Figure 17:**
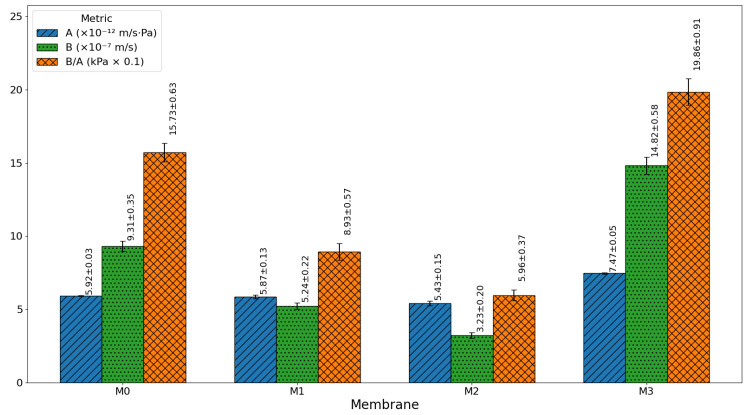
Separation characteristics for Na_2_SO_4_.

The separation performance of the fabricated TFC membranes was thoroughly evaluated based on water permeability, salt rejection, and overall selectivity under different feed conditions. Four salts, NaCl, CaCl_2_, Na_2_SO_4_, and MgCl_2_, were used to study the rejection behavior of monovalent and divalent salts. Varying feed concentrations were applied to replicate real seawater and brackish water desalination conditions. The pristine membrane, M0, served as the control. Three modified membranes, M1, M2, and M3, were created by adding increasing concentrations of GO-PAMAM into the PSF support layer. The desalination characteristics of these membranes are shown in Figure 11, Figure 12, Figure 13, Figure 14, Figure 15, Figure 16 and Figure 17. Together, they give a clear view of how the concentration of GO-PAMAM affects the structure and performance of the membranes.

As shown in Figure 11, NaCl rejection improved significantly with the incorporation of GO-PAMAM into the PSF support layer. The pristine membrane (M0) demonstrated the lowest rejection performance, with rejection values decreasing sharply as feed concentration increased. At a feed concentration of 2000 ppm, M0 achieved a modest rejection of 78.95 ± 0.51%, which dropped substantially to 62.8 ± 1.00% at 20,000 ppm, highlighting its limited ability to exclude salt ions under high TDS conditions. This behavior is typical of unmodified membranes with hydrophobic and non-optimized structures that lack sufficient water-channeling pathways or electrostatic barriers to prevent salt permeation.

Water permeability trends, shown in Figure 12, provide complementary insights into the rejection results. The pristine membrane (M0) exhibited moderate permeability, reaching 2.10 ± 0.05 LMH/bar at 2000 ppm. Its limited hydrophilicity and lack of interconnected water channels restricted water flux, as expected for a standard PSF-based support layer.

With GO-PAMAM addition, water permeability initially increased due to enhanced hydrophilicity and improved substrate porosity. M1 demonstrated a higher permeability (2.39 ± 0.03 LMH/bar) compared to M0, confirming the positive effect of nanoparticle incorporation. The M2 membrane maintained a controlled and stable permeability of 2.13 ± 0.05 LMH/bar, representing the ideal balance between water transport and salt rejection. This indicates that optimal GO-PAMAM loading creates well-defined water pathways without compromising selectivity.

By contrast, the M3 membrane showed the highest permeability, reaching 3.32 ± 0.15 LMH/bar at 2000 ppm. While high water flux might appear advantageous, it coincided with the sharp decline in rejection as observed in Figure 11, suggesting that the additional water transport occurred through non-selective defects rather than functional water channels. This behavior is typical of over-modified membranes, where excessive nanoparticle aggregation disrupts the polyamide layer, resulting in uncontrolled water passage and poor salt exclusion.

The interplay between rejection and permeability across the four membranes is summarized in Figure 13, which directly illustrates the trade-off between water flux and salt exclusion. This trade-off is a fundamental challenge in RO membrane design: improving permeability often compromises selectivity, and vice versa.

M0 demonstrated a moderate permeability but poor rejection, reflecting its unmodified hydrophobic structure. M3, conversely, exhibited a very high permeability but extremely low rejection due to structural defects. The M2 membrane emerged as the optimal configuration, delivering both high rejection and stable permeability. Its superior performance confirms that carefully controlled GO-PAMAM dispersion enables the simultaneous enhancement of water transport and salt exclusion, overcoming the classical permeability–selectivity trade-off. M1 performed moderately well but fell short of M2, indicating that the lower nanoparticle concentration was insufficient to fully optimize membrane morphology.

The study examined how different types of salt affect membrane separation performance using four salts that are often found in brackish water and seawater: NaCl, CaCl_2_, MgCl_2_, and Na_2_SO_4_ [52]. The tests aimed to clarify how specific ions interact with the membrane and how that interaction affects transport and rejection behavior. Figure 14 shows the results for salt rejection, and Figure 15 presents the related water permeability data. These figures reveal that the rejection and permeability patterns change with each type of salt. This variation reflects differences in ionic size, charge, and hydration energy. These salt-related factors, along with the membrane’s structure and surface properties, influence the balance between selective ion rejection and water flow. This study offers valuable insights into the mechanisms that drive desalination performance and provides direction for designing membranes that are more efficient and stable.

As shown in Figure 14, salt rejection follows the order Na_2_SO_4_ > MgCl_2_ > CaCl_2_ > NaCl. A similar trend has been reported by Panahi et al. [18] and Rahimi-Kashkouli et al. [53]. This behavior is mainly attributed to differences in (i) ion valency and (ii) hydrated ion size (and associated diffusivity), which influence both steric hindrance and electrostatic (Donnan) exclusion in the negatively charged PA selective layer. Divalent ions, particularly (SO_4_^2−^, 0.379 nm), experience stronger electrostatic exclusion and greater transport resistance compared with monovalent ions, resulting in higher rejection. Moreover, ions with larger hydrated radii exhibit reduced mobility within the PA network, further increasing their rejection. On the other hand, monovalent chloride ions (Cl^−^, 0.332 nm) face weaker repulsion and are more likely to pass through the membrane. As a result, Na_2_SO_4_ consistently shows the highest rejection rates, while NaCl has the lowest, in line with the Donnan exclusion effect. This mechanism explains why reverse osmosis membranes perform better against multivalent salts; both charge-based exclusion and size-based sieving work together to block the movement of ions. The control membrane (M0) showed consistently low rejection rates for all salts. The rejection for NaCl was only 78.95 ± 0.51%, and for Na_2_SO_4_ it was slightly higher at 81.37 ± 0.61%. These low rates reflect the hydrophobic qualities of the unmodified PSF support layer, which does not have enough water attraction or charged functional groups to support selective ion rejection. In these unoptimized structures, salts can flow through the membrane along uncontrolled paths. This issue may become worse with concentration polarization effects at higher feed salinities.

Incorporating GO-PAMAM nanoparticles into the substrate significantly improved rejection performance. The M2 membrane showed the greatest improvement, achieving 97.11 ± 0.17% rejection for Na_2_SO_4_ and 95.88 ± 0.35% for NaCl, as shown in Figure 14. This impressive performance comes from two main effects:

(1) In TFC RO membranes, salt rejection is governed primarily by the polyamide (PA) selective layer rather than the porous PSF support. Although GO–PAMAM is incorporated within the substrate, it influences rejection indirectly by modifying substrate wettability and surface pore morphology, which promotes a more uniform MPD uptake/distribution and reduces excessive monomer intrusion into substrate pores (funnel effect) during interfacial polymerization. This contributes to the formation of a more continuous and defect-minimized PA layer.
(2) The optimized GO–PAMAM loading in M2 results in a more homogeneous substrate surface, enabling controlled PA growth with fewer imperfections. Consequently, non-selective transport pathways are reduced, leading to higher salt rejection while maintaining favorable water permeability.

However, increasing the GO-PAMAM concentration beyond the optimal 0.06 wt%, as in membrane M3, resulted in a decline in rejection performance for all salts. This decrease is attributed to nanoparticle aggregation, which disrupts the uniformity of the PSF support and creates localized defects in the PA layer. These defects act as non-selective transport channels, allowing salts to permeate freely. Therefore, with a higher content of nanoparticles, M3 had a lower rejection than M2 for all salts, stressing the importance of correct nanoparticle optimization to prevent the chances of performance deterioration because of overloading. The decreased rejection is probably due to the formation of non-uniform polyamide layers that contain larger defects. These defects, when becoming larger than the hydration shells of multivalent ions like Mg^2+^ and SO_4_^2−^, provide non-selective pathways, allowing ions of both monovalent and multivalent nature to pass through. This lack of selectivity may accelerate performance decline due to the increased non-selective salt transport through defects and reduced integrity of the selective layer.

The water permeability results shown in Figure 15 indicate an inverse correlation with salt rejection, illustrating the classical permeability–selectivity trade-off characteristic to RO membranes. For the pristine membrane (i.e., M0), water permeability was low through all salt solutions because of its generally hydrophobic nature and less optimal structure for water transport (NaCl: 2.10 ± 0.05 LMH/bar; CaCl_2_: 2.07 ± 0.04 LMH/bar; Na_2_SO_4_: 2.05 ± 0.09 LMH/bar; MgCl_2_: 2.07 ± 0.08 LMH/bar). Upon GO-PAMAM incorporation, the water flux increased initially (i.e., M1) due to the enhancement in hydrophilicity and pore connectivity of the support layer, which facilitated the passage of water. For M1, flux enhancement was observed for all salt solutions but varied slightly with respect to the type of salt present (NaCl: 2.39 ± 0.03 LMH/bar; CaCl_2_: 2.27 ± 0.01 LMH/bar; Na_2_SO_4_: 2.11 ± 0.05 LMH/bar; MgCl_2_: 2.25 ± 0.01 LMH/bar). The lower flux for solutions containing multivalent salts such as Na_2_SO_4_ and MgCl_2_ is due to their high osmotic pressure and the large size of hydrated ions, which increase hindrance for water transport through the membrane.

M2 displayed the most balanced performance for water permeability across all salts (NaCl: 2.13 ± 0.05 LMH/bar, CaCl_2_: 1.98 ± 0.02 LMH/bar, Na_2_SO_4_: 1.92 ± 0.05 LMH/bar, MgCl_2_: 1.93 ± 0.01 LMH/bar). In contrast, M3 exhibited an excessively high water flux for all salt solutions (NaCl: 3.32 ± 0.15 LMH/bar, CaCl_2_: 3.29 ± 0.04 LMH/bar, Na_2_SO_4_: 2.69 ± 0.02 LMH/bar, MgCl_2_: 3.12 ± 0.00 LMH/bar). This surge in flux again signals uncontrolled water transport caused by structural defects and non-uniform polyamide layer formation due to nanoparticle overloading. While a higher water flux may seem beneficial, it corresponds to a reduced selectivity, as these defects allow both water and salts to pass through non-selective pathways, ultimately diminishing desalination performance and efficiency.

The performance of M2 further indicates that optimizing nanoparticle concentration is essential for improving overall membrane performance. At this concentration, GO-PAMAM provides sufficient hydrophilic sites and structural uniformity to form a compact, defect-free polyamide layer, ensuring a high water flux and high salt rejection. In contrast, excessive loading, as in M3, disrupts this balance and leads to defect formation, which aligns with the mechanistic explanation discussed earlier.

The findings in Figure 14 and Figure 15 have significant practical implications for the design of next-generation RO membranes. The ability of M2 to maintain high rejection and stable water permeability across a range of salt types demonstrates its suitability for diverse feedwaters, including brackish water and seawater desalination. While concentration polarization was not directly quantified in this work, the stable rejection–permeability behavior indicates that substrate modification can regulate selective-layer formation and transport performance under the applied cross-flow conditions. Under-modified membranes like M1 provide only partial improvements, whereas over-modified membranes like M3 suffer from compromised structural integrity and performance losses.

The synergy between graphene oxide’s mechanical robustness and PAMAM’s functional versatility positions GO-PAMAM hybrids as an effective platform for advanced membrane engineering. Beyond desalination, these membranes hold promise for wastewater treatment, industrial effluent purification, and selective ion recovery.

Figure 16 and Figure 17 present the water permeability (A), salt permeability (B), and salt-to-water permeability ratio (B/A) for the fabricated membranes, providing a clear understanding of the trade-off between permeability and selectivity. For NaCl (Figure 16), the pristine membrane (M0) showed a high salt permeability (31.12 ± 0.41 × 10^−7^ m/s) and a very high B/A ratio (53.35 ± 1.64), indicating a weak salt rejection and poor separation efficiency. The introduction of 0.03 wt% GO-PAMAM (M1) significantly improved performance, lowering salt permeability to 12.46 ± 0.31 × 10^−7^ m/s and B/A to 18.74 ± 0.65, due to enhanced surface hydrophilicity and improved polyamide layer formation. The M2 membrane (0.06 wt% GO-PAMAM) achieved the best balance, with the lowest salt permeability (5.10 ± 0.50 × 10^−7^ m/s) and B/A ratio (8.60 ± 0.75), reflecting superior salt rejection while maintaining stable water permeability. However, with further nanoparticle loading at 0.10 wt% (M3), salt permeability increased sharply (30.54 ± 7.58 × 10^−7^ m/s) and B/A rose to 33.73 ± 9.72, highlighting the structural defects caused by nanoparticle aggregation that created non-selective pathways for salt transport.

For Na_2_SO_4_ (Figure 17), a similar trend was observed but with generally lower salt permeability values due to the higher charge and larger hydrated radius of sulfate ions. The pristine membrane (M0) again performed poorly, while M1 showed noticeable improvement. M2 provided the most efficient separation, with salt permeability reduced to 3.23 ± 0.20 × 10^−7^ m/s and a very low B/A ratio (5.96 ± 0.37), indicating improved desalination performance. In contrast, M3 exhibited a higher water permeability but also increased salt passage, signaling instability and a loss of selectivity at high nanoparticle concentrations.

These results indicate that an optimal GO–PAMAM concentration of 0.06 wt% (M2) provides a favorable balance between high water flux and salt rejection.

### 3.7. Organic Fouling of the Prepared Membranes

**Figure 18:**
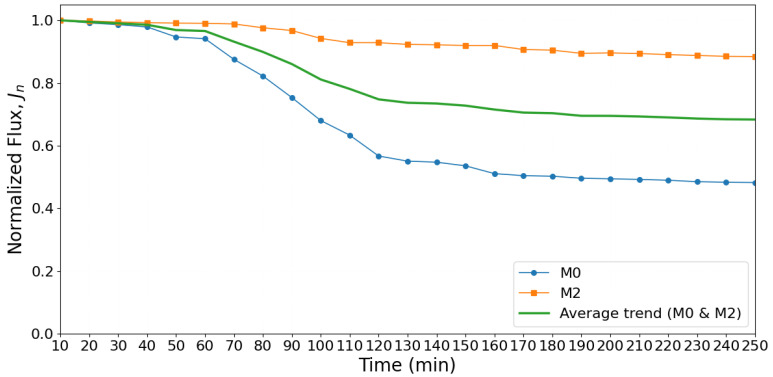
Normalized flux decline over 240 min showing severe fouling for M0 and stable performance for M2.

**Figure 19:**
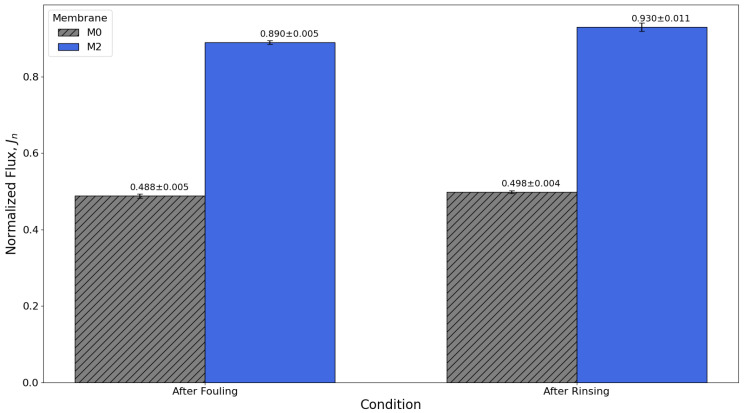
Flux recovery after hydraulic rinsing, with M2 showing reversible fouling and M0 dominated by irreversible fouling.

The fouling resistance and cleaning efficiency of the prepared membranes were evaluated through time-dependent flux decline and recovery experiments, as illustrated in Figure 18 and Figure 19. The normalized flux (J_n_) was used to monitor membrane performance over time and to account for differences in initial pure water flux (J_0_). This normalization allows a direct comparison between membranes under identical operating conditions, highlighting their fouling behavior more accurately.

Figure 18 shows the progression of normalized flux over 250 min of continuous filtration for the pristine membrane (M0) and modified membrane (M2). The pristine membrane exhibited a sharp and rapid decline, with normalized flux decreasing from 1.0 to approximately 0.48 by the end of experiment. The observed decline suggests severe fouling, which can be attributed to foulant–membrane interactions leading to compact foulant layer formation and pore blockage [54]. Such behavior is typical for hydrophobic membranes with heterogeneous and irregular surface morphologies, where rough regions and defects act as nucleation sites for foulant accumulation. As fouling progresses, hydraulic resistance rises, significantly reducing water permeability and overall process efficiency.

In contrast, M2 demonstrated a slower flux decline and reached a normalized flux of 0.89 after 250 min, indicating improved fouling resistance [55]. AFM analysis showed that M2 exhibited a slightly higher surface roughness than M0; however, SEM images revealed a more uniform polyamide layer with reduced structural defects. Such selective-layer uniformity may reduce localized sites where foulant deposition preferentially initiates and limits the formation of defect-driven transport pathways. It should be noted that antifouling behavior is governed by multiple surface characteristics, including hydrophilicity, surface charge, and surface morphology/roughness, rather than roughness alone [56,57]. Under cross-flow conditions, the improved surface properties of M2 can weaken foulant–membrane adhesion, facilitating the removal of deposited foulants by hydrodynamic shear and thereby reducing the extent of irreversible fouling.

The performance contrast between M0 and M2 has direct implications for operational stability. The rapid flux decline in M0 suggests that frequent chemical cleaning would be required to maintain performance, increasing operating costs and reducing membrane lifespan. In comparison, M2’s gradual decline reflects predictable fouling behavior, enabling longer operation cycles and improved sustainability for desalination processes.

Figure 19 compares flux recovery after a simple hydraulic rinse. M0 recovered only about 50% of its initial flux, suggesting predominantly irreversible fouling, likely associated with foulant adhesion and penetration into the membrane pores. In contrast, M2 achieved a 93% recovery, showing that fouling was mostly reversible and consisted of weakly bound deposits easily removed through physical rinsing.

These results confirm that 0.06 wt% GO-PAMAM significantly enhances antifouling performance by preventing foulant adhesion and promoting efficient foulant detachment. Combined with its superior salt rejection and water permeability, M2 demonstrates the potential to deliver stable long-term operation with reduced cleaning requirements and lower maintenance costs, positioning it as a promising candidate for next-generation sustainable reverse osmosis membranes.

### 3.8. Comparison of the Prepared Membrane with Some Reported Membranes

RO membrane development for a better desalination efficiency through an increased rate of water permeation and rejection of salt has been a subject of research for many years. Table 2 summarizes representative studies and presents reference points to contrast them against the membrane optimized in this study (M2, 0.06 wt% GO-PAMAM). Since the membranes have been tested under varied assuming conditions like applied pressure, concentration of feed, and water chemistry, the table serves as a general visualization of performance trends and not a direct comparison.

The M2 membrane exhibits a high desalination performance, achieving a water flux of 42.63 LMH at 20 bar and 2000 ppm feed concentration, while maintaining high rejection values for all tested salts: 97.10% for Na_2_SO_4_, 96.01% for CaCl_2_, 96.19% for MgCl_2_, and 95.88% for NaCl. These results surpass or closely rival those of other advanced membranes, including nanocomposite thin-film membranes incorporating materials such as GO-SiO_2_, TiO_2_-GO, and multi-walled carbon nanotubes (MWCNTs).

It is noteworthy that some reported membranes exhibit high rejection but at considerably lower operating pressures or for limited salt types, making direct numerical comparisons challenging. For instance, membranes tested at 6–10 bar with 1000 ppm feed often display reduced water flux due to lower driving forces. In contrast, the M2 membrane achieves both a high rejection and competitive flux under the more demanding conditions of 20 bar and 2000 ppm, demonstrating its robustness and suitability for practical desalination applications.

## 4. Conclusions

In this study, TFC polysulfone RO membranes were fabricated by incorporating a GO–PAMAM nanocomposite into the substrate at three loadings (0.03, 0.06, and 0.10 wt%), in addition to a pristine control membrane. The GO–PAMAM nanocomposite and the fabricated membranes were characterized using SEM, AFM, FTIR, XRD, and contact angle measurements to evaluate their structural, chemical, and surface properties. The results showed that the incorporation of GO–PAMAM improved membrane hydrophilicity, where the contact angle decreased from 71° (M0) to 60° (M2), corresponding to a reduction of approximately 15.5%. Among the membranes, the M2 membrane (0.06 wt% GO–PAMAM) provided the best overall performance, achieving a water flux of 42.6 L m^−2^ h^−1^ and NaCl rejection of 95.88% at 20 bar and a feed flow rate of 4 L min^−1^. The optimized membrane also showed improved antifouling behavior, retaining 93% of its initial flux after fouling. However, further increasing GO–PAMAM loading to 0.10 wt% resulted in reduced performance due to nanoparticle aggregation and the formation of structural non-uniformities. Based on the overall results, a GO–PAMAM loading of 0.06 wt% can be considered the optimum concentration for the substrate modification of TFC RO membranes.

## Figures and Tables

**Table 1:** Composition of PSF casting solution and GO–PAMAM loading used for TFC membrane fabrication.

Membrane	PSF (wt%)	DMF (wt%)	SDS (wt%)	MPD (wt%)	TMC (wt%)	GO-PAMAM (wt%)
M0	17.5	82.5	0.2	3	0.1	0
M1	17.5	82.47	0.2	3	0.1	0.03
M2	17.5	82.44	0.2	3	0.1	0.06
M3	17.5	82.40	0.2	3	0.1	0.1

**Table 2:** Comparison of the optimized GO-PAMAM membrane (M2) with reported RO membranes under varying operating conditions.

Membrane Type	Test Condition	Maximum Flux (LMH)	R_Na2SO4_ (%)	R_CaCl2_ (%)	R_MgCl2_ (%)	R_NaCl_ (%)	Ref.
TFN-0.05_CNCs_	10 Bar, 1000 ppm	22.47	93.44	71.03	79.72	62.68	[53]
TFC-1.0_BDSA_	6 Bar, 1000 ppm	24	95.4	-	88.6	92.5	[58]
TFN-50_Silica_	6 Bar, 1000 ppm	42.6	98.3	-	67.2	42.6	[59]
PSF-0.5_G0-SiO2_	12 Bar, 2000 ppm	44.44	97.01	88.29	93.33	81.44	[18]
PA/MWCNT_s_ 0.01%	20 bar, 2000 ppm	41	-	-	-	92	[60]
PA/TiO_2_-GO	10 bar, 2000 ppm	63.3	97	-	-	97	[61]
PSF-0.06_GO-PAMAM_	20 bar, 2000 ppm	42.628	97.10	96.01	96.19	95.88	This study

## Data Availability

The original contributions presented in this study are included in the article. Further inquiries can be directed to the corresponding author.

## Nomenclature

J_w_	Water flux (LMH, L·m^−2^·h^−1^)
ΔV	Volume of permeate collected (L)
Am	Effective membrane area (m^2^)
Δt	Time of collection (h)
A_w_	Water permeability coefficient (LMH/bar)
ΔP	Applied transmembrane pressure (bar)
R	Salt rejection (%)
C_f_	Feed salt concentration (ppm)
Cp	Permeate salt concentration (ppm)
B	Salt permeability coefficient (m/s)
B/A	Salt-to-water permeability ratio (dimensionless)
J_0_	Initial pure water flux before fouling begins (LMH)
J(t)	Instantaneous permeate flux at time t (LMH)
Jn	Normalized flux at time t (dimensionless)
R_a_	Average surface roughness measured by AFM (nm)

## Abbreviations

AFM	Atomic Force Microscopy
FTIR	Fourier Transform Infrared Spectroscopy
BSA	Bovine Serum Albumin
DMF	Dimethylformamide
SEM	Scanning Electron Microscopy
GO	Graphene Oxide
PAMAM	Poly(amidoamine)
IP	Interfacial Polymerization
LMH	Liter per Square Meter per Hour (L·m^−2^·h^−1^)
MPD	m-Phenylenediamine
NaCl	Sodium Chloride
PA	Polyamide
PSF	Polysulfone
RO	Reverse Osmosis
TFC	Thin-Film Composite
TMC	Trimesoyl Chloride
XRD	X-ray Diffraction
